# Mediation role of alexithymia, sensory processing sensitivity and emotional-mental processes between childhood trauma and adult psychopathology: a self-report study

**DOI:** 10.1186/s12888-021-03532-4

**Published:** 2021-10-15

**Authors:** Pelin Karaca Dinç, Seda Oktay, Ayşegül Durak Batıgün

**Affiliations:** 1 Ankara University, Ankara, Turkey; 2 Department of Psychology, Faculty of Languages History and Geography, University of Ankara University, No:45-45/A 06100, Sıhhiye, Ankara, Turkey

**Keywords:** Alexithymia, Mentalization, Psychopathology, Sensory processing, Trauma

## Abstract

**Background:**

There is overwhelming evidence for a strong association between childhood trauma and adult psychopathology. This study aimed to investigate the mediation roles of alexithymia, sensory processing sensitivity, and emotional-mental processes in the relationship between childhood traumas and adult psychopathology.

**Methods:**

The sample consisted of 337 people (78.9% female, 21.1% male) aged between 20 and 64 years. Participants filled the scales online via a Google form. Reading Mind in the Eyes (EYES), Sensory Processing Sensitivity Scale (SPS), Toronto Alexithymia Scale (TAS-26), Childhood Trauma Questionnaire (CTQ), and the Brief Symptom Inventory (BSI) were used. PROCESS (Model 4) macro was used to examine the mediating role of sensory processing sensitivity, alexithymia, and the EYES test results in the relationship between childhood trauma and psychopathology.

**Results:**

The results of mediation analysis demonstrated that sensory processing sensitivity and alexithymia mediated the relationship between childhood trauma and adult psychopathology. However, the EYES test (mentalization) did not mediate in this relationship.

**Conclusion:**

This study shows that childhood traumas may relate to more psychological symptoms in individuals with high sensory processing sensitivity and alexithymia. Our study may contribute to the understanding of what may lead to a person’s vulnerability to experiencing psychological symptoms after childhood trauma. It may be crucial that future treatment and intervention programs should include sensory sensitivity and alexithymia. Sensory processing sensitivity and alexithymia can be examined in the treatment of psychological problems of individuals who have experienced childhood trauma.

## Introduction

Childhood trauma is defined as a psychological consequence of sudden or ongoing disruptive experiences that temporarily leave the child feeling helpless, damage coping mechanisms, or a series of continuous external injuries [1]. Bernstein and Fink [2] proposed a definition about child abuse and neglect, which includes verbal attacks on a child’s sense of self-worth, physical attacks that pose a risk of injury, sexual contact with a child, failure to provide basic psychological and emotional needs and to meet basic needs. There is broad evidence that childhood traumas are frequent in various populations. For instance, in a study conducted with a German sample, 2510 female participants aged 14–94 completed the Child Trauma Questionnaire (CTQ); as a result, 2.6% of the participants stated that they were subjected to severe emotional abuse, 3.3% physical abuse, 2.3% sexual abuse, 7.1% emotional neglect, and 9% physical neglect [3]. In a meta-analysis of mainly USA and Canada non-clinical samples under 18 years of age, physical and emotional neglect were prevalent in 16.3 and 18.4%, respectively [4]. Consistently, in a meta-analysis of 65 studies involving participants from 22 countries, results indicated that 7.9% of men and 19.7% of female were exposed to sexual abuse before the age of 18 [5]. In another study, 32.3% of Turkish students stated that they experienced emotional abuse, 14.6% physical abuse, and 8.9% sexual abuse [6]. This situation reveals that sexual abuse is a universal problem [7]. Getting knowledge of childhood trauma and other forms of violence is difficult due to social taboos; studies show that traumatic childhood experiences are prevalent [8]. In addition to the prevalence of these experiences, traumatic childhood experiences have developmental and long-term effects in many areas of functionality [8].

Childhood traumas can interrupt developmental processes and lead to neurological, physiological, and psychological consequences [9]. Exposure to childhood trauma is a potential risk factor for psychopathology in adulthood [10]. Adults who experience childhood trauma develop many psychopathologies, such as mood disorders [11] and anxiety disorders [12]. Childhood trauma also increases the likelihood of post-traumatic stress disorder (PTSD) [13], obsessive-compulsive disorder [14], personality disorders [15], and psychotic symptoms [16]. Individuals with childhood trauma have a higher risk of suicide, suicide attempts and self-harming behaviors [17]. Also, childhood trauma is associated with alcohol and substance abuse in adulthood [18]. In addition to these, traumatic experiences in childhood are also closely related to physical symptoms, such as chronic fatigue [19], pain disorders [20], sleep problems [21], and cognitive impairment [22] in adulthood.

Stress is defined as stimuli or experiences that cause adverse emotional reactions or feelings, such as fear and loss of control [23]. The increased risk for the development of psychopathology can be better understood with reflection upon how the body responds to stressful experiences. The disruption of the hypothalamic-pituitary-adrenal axis in response to stress is one pathway to psychopathology. Maltreatment from parents in early childhood is one of the most important sources of stress [23]. Childhood trauma is associated with an abnormality in HPA axis responsiveness to stress [24]. In a study with young people exposed to different types of childhood trauma, it was stated that hyper or hypo reactive HPA increased the internalization and externalization of symptoms [25].

The relationship between alexithymia [26], sensory sensitivity [27], emotion recognition skills [28] and cortisol have been studied in the literature. Studies indicated that alexithymia was associated with elevated cortisol levels [29]. Similarly, high cortisol levels appeared to be associated with sensitivity in sensory processing [27]. The relationship between the ability to recognize emotions and cortisol level is different. Although some studies have indicated that individuals who are good at recognizing emotions have high cortisol levels [28], some have not reported a relationship between emotion recognition and cortisol levels [30]. Individuals may have difficulty in recognizing emotions regardless of cortisol level. Healthy men and women were divided into two groups, one of which was given a placebo, the other cortisol to examine the effect of cortisol on empathy and emotion recognition. There was no significant association between the increase in cortisol level and the recognition and empathy of the senses [30]. Smeets and his colleagues [31] also found no significant relationship between change in cortisol levels and facial recognition skills. Being unable to read emotions, regardless of cortisol level, may relate to more anxiety and stress. In a study conducted with children, those with anxiety disorders exhibited less skill in perceiving facial expressions of adults compared to the healthy group [32]. Considering the impact of trauma on the HPA axis (cortisol levels), it is thought that trauma causes a significant deterioration in cognitive, executive functions, emotional skills, and sensitivity to stress, which relates possible pathways to psychopathology. Therefore, possible contributing factors of childhood trauma to relate psychological distress might be emotional and mental processes (EYES test), alexithymia associated with skills in understanding and expressing emotions, and sensory processing sensitivity associated with sensitivity to stimuli.

The concept of alexithymia has been introduced by Sifneos, and it means that there is no word for emotions [33]. Individuals with alexithymia have difficulty in recognizing their own emotions. These individuals have reality-based cognitive styles, weak emotional and imaginative experiences [34]. Individuals with childhood trauma had a higher likelihood of alexithymia levels [35] In addition, studies have demonstrated that individuals with higher levels of alexithymia also develop more psychological symptoms such as somatization [36], alcohol-related problems [37], posttraumatic stress disorder [38], depression [39] and eating disorders [40].

The term mentalizing, one of the cognitive and emotional functions, refers to the process in which inferences are made about mental states [41]. People’s faces become an important resource for inference [41]. Children with trauma have difficulties in understanding and recognizing emotions from facial expressions [42]. In addition, studies have revealed that people with psychological symptoms in adulthood have difficulty in recognizing emotions from facial expressions [43, 44].

Sensory processing sensitivity, thought to be another possible contributing factor, is a term introduced by Aron and Aron [45] that defines the individual sensitivity to the perception of social and emotional stimuli against internal and external stimuli. For instance, the individual is sensitive to internal body signals or sensory stimuli such as pain, hunger, and to external/environmental sensory stimuli such as loud sounds and sharp odors. The fact that the individual perceives and interprets intensively such stimuli faster and responds faster to these stimuli indicates that the individual has a high sensitivity to sensory processing [45]. There are studies on the biomarker of sensory processing sensitivity. Children with sensory processing disorder may have decreased parasympathetic nervous system activities compared to normally developing children [46, 47], and these children exhibit poor adaptive behaviors in communication and daily life [47]. In the study investigating the relationship between abuse, neglect, and maltreatment in adopted children and sensory domains, it was pointed out that sensory processing deficits differ according to the type of maltreatment [48]. It has been found that children with a history of abuse tend to be hyper-responsive to sensory input, while children who experience neglect are under-responsive [48]. In addition, there are studies in the literature showing that the pain threshold of individuals with childhood trauma decreases and their sensitivity to stimuli increases in adulthood [48, 49]. Individuals with increased sensitivity to stimuli and a high level of sensory processing sensitivity are at increased risk of developing various psychological problems, including stress, anxiety, depression [50], obsessive-compulsive disorder [51], and sleep problems [52] since abnormalities in sensory processing and sensitivity cause impairments in daily functioning and may serve as an indicator of mental disorders [53].

The relationship between alexithymia and childhood trauma and psychopathology has been studied extensively (e.g. [54, 55]). The number of studies examining all childhood trauma, psychopathology, sensory processing sensitivity, emotional-mental processes (EYES test) and alexithymia together were limited, although there are studies in which the relationship between the variables and psychopathology were examined separately (e.g. ( [32, 36, 43, 50])). In this context, this study aims to present up-to-date and comprehensive information to the literature by considering all these variables together. The current findings may serve to understand the predisposing factors in the relationship between childhood trauma and psychopathology. Accordingly, it has been hypothesized that emotional-mental processes, sensory processing sensitivity, and alexithymia would mediate the relationship between childhood traumatic experiences and adult psychopathology.

## Methods

### Participants

In this study, firstly, 374 individuals between the ages of 20–64 were reached through the convenience sampling technique. Appropriate sampling was first accessed by the researchers around the campus. Hand-held questionnaires were administered to 35 volunteer participants around the campus. Later, online data were collected via a Google form to reach more people and make it easier for researchers. In the data collected via the paper-and-pencil method, more than half of the scale items were empty, and 35 participants were excluded from the study. Only online data was used. Also, 2 participants under the age of 20 were excluded from the online data. Age limit of 20 years and above was determined as an inclusion criterion because “These questions are about some events that may have happened to you in your childhood and early adolescence (before the age of 20)” direction was included in the Turkish version of CTQ. For this reason, we set the age limit of 20 for the participants. In the online form, filling out all the questionnaires was marked as mandatory, so there was no missing data. In the Informed Consent Form, the participants were already given the opportunity to withdraw from the study at any time. At the last stage, 337 people aged between 20 and 64 (M = 29.89, SD = 10.53) were included in the analysis. 78.9% (*N* = 266) of the participants were females and 21.1% (*N* = 71) were males. 58.8% of participants (*N* = 198) stated their perceived socio-economic levels as moderate, 32.9% (*N* = 111) good, 1.5% (*N* = 5) very good, 6.2% (*N* = 21) bad, 0.6% (*N* = 2) very bad. 95% of the participants (*N* = 320) were university graduates, 4.2% (*N* = 14) were high school graduates, 0.6% (*N* = 2) were secondary school graduates, and 0.3% (*N* = 1) were primary school graduates.

### Instruments

#### Demographic information form

This form contained sociodemographic information about age, gender, education, and socioeconomic levels.

#### Emotional and mental processes (EYES test)

Reading mind in the eyes test (EYES) developed by Baron-Cohen and his colleagues [56] was used to evaluate emotional and mental processes. Participants are shown a black and white photograph of eyes and that the four options are emotion words, with one word best describing the emotion displayed in the photograph. There are four options, one correct answer and three distractions for each image. The Turkish version of the test consists of 32 questions. The reliability results of the test with Kuder –Richardson 20 were found as KR20 = 0.72 [57]. In this study, Cronbach’s alpha value was found 0.47. Higher scores on the scale show the person’s high-level ability to recognize emotions and expressions by looking at the eyes.

#### Sensory processing sensitivity scale (SPS)

The Sensory Processing Sensitivity Scale (SPS) (Highly sensitive person scale) developed by Aron and Aron [45] was used to measure individuals’ differences in processing both internal (e.g., pain, hunger) and external (e.g., art, noise, emotional states of others) stimulus. This scale consisting of 27 items is scored in the 7-point Likert type. The Turkish version of the scale had a four-factor psychometric structure with high internal consistency [58]. These factors are called Overstimulation Sensitivity, External Stimulus Sensitivity, Aesthetic Sensitivity, Harm Avoidance. In this study, the Cronbach alpha value for the total score is 0.87. Higher scores indicate higher levels of sensory processing sensitivity.

#### Alexithymia (TAS-26)

Toronto Alexithymia Scale (TAS-26) was used to measure the level of alexithymia. TAS-26 was developed by Taylor and his colleagues [34]. TAS-26 is a 26-item with a 5-point Likert-type scale consisting of 4 subscales. In the Turkish validity study conducted by Motan and Gençöz [59], it was observed that the scale had 3 factors. Factors were named as Difficulty in Emotional Communication, Difficulty in Recognizing and Defining Emotions, Lack of Imagination. In this study, the Cronbach alpha value for the total score is 0.80. Higher scores indicate higher levels of alexithymia.

#### Childhood trauma (CTQ)

Childhood Trauma Questionnaire (CTQ), developed by Bernstein and his colleagues [2], was used to evaluate childhood traumas. The questionnaire, adapted to Turkish by Şar and his colleagues [60], has subscales of sexual, physical, emotional abuse and physical and emotional neglect. The scoring of the scale, which evaluates the abuse and neglect experiences before the age of 20 based on self-report, is 5-point Likert type. In the study of the Turkish version of the scale, the Cronbach alpha for the subscales of the questionnaire were found as 0.73 for sexual abuse, 0.90 for physical abuse, 0.90 for emotional abuse, 0.77 for physical neglect, and 0.85 for emotional neglect [60]. In this study, the Cronbach alpha value for all items of the scale is 0.91. Higher scores on the scale indicate the excess of traumatic life in childhood.

#### Psychopathology (BSI)

Brief Symptom Inventory (BSI) was originally developed by Derogatis [61]. It consists of 53 items and is based on self-report. It was used to evaluate psychopathology. In the Turkish adaptation study conducted by Şahin and Durak [62], subscales were named as depression, anxiety, negative self, somatization and hostility. The Cronbach alpha values obtained for the subscales ranged between 0.63 and 0.86. In this study, the Cronbach alpha value for the total score is 0.97. Higher scores indicate the excess of psychopathology.

#### Procedure

Ethical permission was obtained from Ankara University Human Research Ethics Committee. Necessary permissions were obtained from the researchers who adapted the scales to Turkish for the use of the scales. The printed version of the scales and pens were provided by the researchers so that the participants around the campus could fill out the questionnaires. At the same time, online data was collected via a Google form to reach more people. Participants in the study are expected to read and approve the Informed Consent Form before participation. They participated voluntarily and were not given an incentive or reward for participating in the study. Participants had the right to leave without any pressure at any stage of the study. Each scale was administered to the participants in the same order. It took approximately 50 min to complete the questionnaires.

#### Data analysis

Statistical analyses were carried out via Statistical Package for Social Sciences – SPSS, version 21.00. It was used to conduct descriptive analysis (age, gender, education) and comparison of mean scores of CTQ and BSI. Second, the relationship between the study variables were investigated using Pearson’s correlation analysis. Gender was controlled in the mediation analysis and the Bootstrap method with 1000 samples was applied to examine the mediating role of sensory processing sensitivity, alexithymia, and the EYES test results in the relationship between childhood trauma and psychopathology via the SPSS PROCESS (Model 4) [63].

## Results

The subscales of CTQ and BSI scale were examined in terms of demographic variables (gender, education, perceived socio-economic level). Depression and somatization, which are subscales of the BSI, significantly differ in terms of only gender. Except for gender, there is no significant difference between demographic variables and subscales. Depression scores of females are higher than males t(335) = 2.70, *p* < .01. Somatization scores of females are higher than males t(335) = 2.28, p < .01.

The relationships between the total scores obtained from all scales and demographic variables were examined with One-Way ANOVA and Independent samples T-test. There is no significant difference between the total scores of all scales according to education level and perceived socio-economic level. There are significant differences in some of the total scores of the scales in terms of only gender. Descriptive statistics and comparisons by gender (t-tests) for the study variables are presented in Table 1.

**Table 1:** Descriptive statistics and t values according to gender

	Gender					
	Female (*N* = 266)		Male (*N* = 71)			
	M	SD	M	SD		
CTQ	39.43	14.75	39.86	16.34	−0.21	
TAS-26	59.59	12.66	58.34	14.32	0.72	
SPS	128.25	21.03	119.22	19.03	3.27^***^	
EYES	24.43	2.95	23.22	3.71	2.89^**^	
BSI	119.47	41.82	109.84	42.30	1.72	

*Note* 1. *BSI* Brief Symptom Inventory, *SPS* Sensory Processing Sensitivity Scale, *TAS-*26 Toronto Alexithymia Scale, *CTQ* Childhood Trauma Questionnaire, *EYES* Reading Mind in The Eyes Test.

*Note* 2. ^***^ *p* < .001, ^**^ *p* < .01, ^*^ *p* < .05

### Partial correlations between all variables

Considering that gender may be a confounding variable, gender was controlled in the correlation analysis. CTQ, SPS, BSI, TAS-26 total scores are positively associated with each other. Table 2 shows that the EYES test results do not significantly correlate with the CTQ, SPS, BSI, TAS-26 results.

**Table 2:** Partial Correlations Between All Variables

	CTQ	TAS-26	SPS	EYES	BSI	
CTQ	–					
TAS-26	0.33^***^	–				
SPS	0.24^***^	0.20^***^	–			
EYES	−0.08	−0.09	0.05	–		
BSI	0.05^***^	0.57^***^	0.47^***^	0.04	–	

*Note* 1. *BSI* Brief Symptom Inventory, *SPS* Sensory Processing Sensitivity Scale, *TAS-*26 Toronto Alexithymia Scale, *CTQ* Childhood Trauma Questionnaire, *EYES* Reading Mind in The Eyes Test.

*Note* 2. ^***^ *p* < .001, ^**^ *p* < .01, ^*^ *p* < .05

*Note* 3. The results were obtained after controlling for the effect of the gender variable

### Mediating role of sensory processing sensitivity, alexithymia, emotional-mental processes (EYES) in the relationship between childhood trauma and psychopathology

Childhood trauma is positively associated with alexithymia (β =0 .28, t = 6.34, *p* < .001, 95% CI [0.20, 0.37]). Childhood trauma is positively associated with sensory processing sensitivity (β = 0.33, t = 4.59, p < .001, 95% CI [0.19, 0.48]). Childhood trauma is not associated with the EYES test (β = − 0.02, t = − 1.49, *p* = .14, 95% CI [−.04, 0.01]).

Alexithymia is positively associated with psychopathology (β = 1.41, t = 10.65, p < .001, 95% CI [1.15, 1.67]). Sensory processing sensitivity is positively associated with psychopathology (β = 0.65, t = 7.92, p < .001, 95% CI [0.49, 0.81]). The EYES test is positively associated with psychopathology (β = 1.14, t = 2.19, *p* < .05, 95% CI [0.11, 2.17]).

**Fig. 1:**
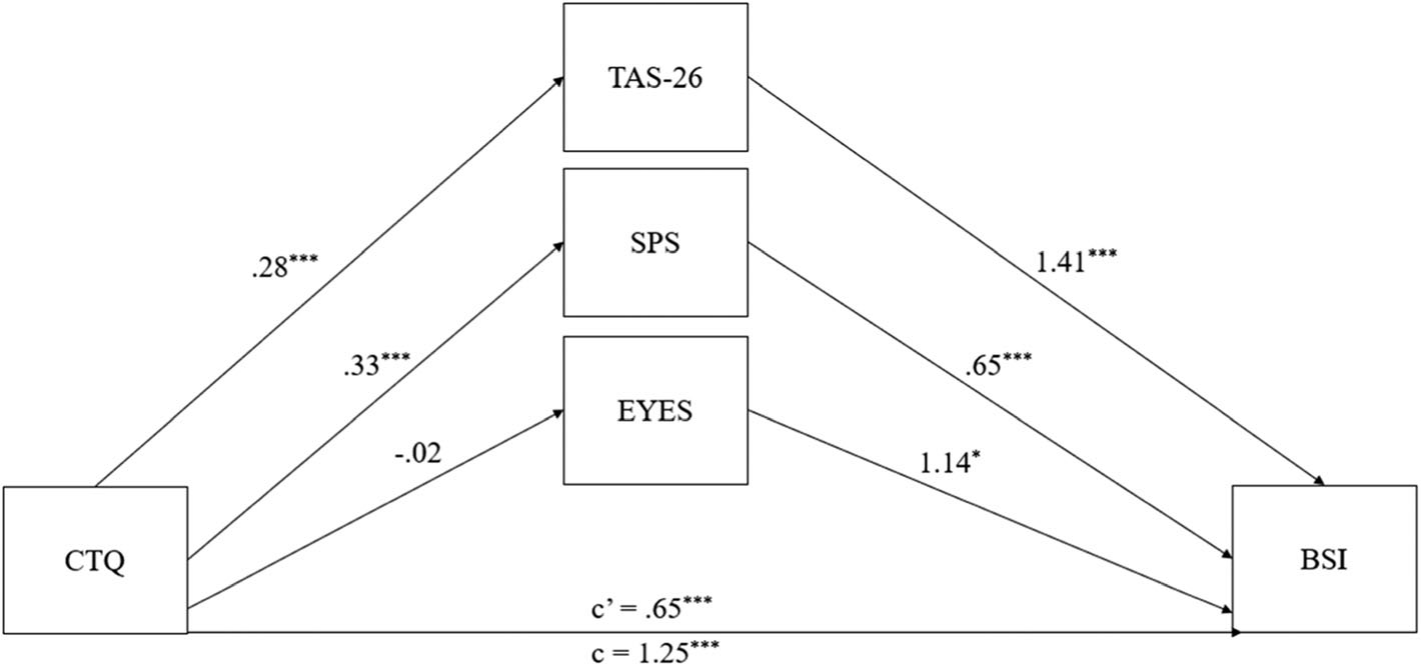
Mediating role of Sensory processing sensitivity, Alexithymia, Eyes in the relationship between childhood trauma and psychological symptoms. Note 1. BSI = Brief Symptom Inventory, SPS = Sensory Processing Sensitivity Scale, TAS-26 = Toronto Alexithymia Scale, CTQ = Childhood Trauma Questionnaire, EYES = Reading Mind in The Eyes Test. Note 2 ^***^ *p* < .001, ^**^ *p* < .01, ^*^ *p* < .05. Note 3. The results were obtained after controlling for the effect of the gender variable

Mediation analysis was performed to examine the mediator roles of sensory processing sensitivity, alexithymia, and EYES test results in the relationship between childhood trauma and psychopathology by controlling the gender variable (see Fig. 1).

According to the results, the indirect effects of both alexithymia (b = 0.40, boot SE = 0.07, 95% CI [0.25, 0.54]) and sensory processing sensitivity (b = 0.22, boot SE = 0.05, 95% CI [0.13, 0.32]) on the relationship between childhood trauma and psychopathology are significant. The indirect effect of the EYES test on the relationship between childhood trauma and psychopathology is not significant (b = − 0.02, boot SE = 0.02, 95% CI [− 0.06, 0.01]). Accordingly, sensory processing sensitivity and alexithymia significantly mediate the relationship between childhood trauma and psychopathology.

The direct effect of childhood trauma on psychopathology was found as .65 (β = 0.65, t = 5.65, *p* < .001, 95% CI [0.43, 0.88]), and the total effect as 1.25 (β = 1.25, t = 9.20, p < .001, 95% CI [0.98, 1.52]). Considering the whole model, the model is significant and explains 51% of the variance (R^2^ = 0.51, F (5, 331) = 70.01, *p* < .001).

## Discussion

In this study, variables that are thought to mediate the association of childhood trauma on psychopathology were examined. Childhood trauma positively predicted psychopathology, and the sensory processing sensitivity and alexithymia had a mediating role in this association.

According to the literature, childhood traumas can be a predisposition or risk factor for different psychopathology (e.g. ( [11, 16, 18, 20])). In this study, the relationship between childhood traumas and psychopathology (depression, anxiety, negative self, somatization and hostility) was supported, and it was seen that the two variables mediating this relationship were sensory processing sensitivity and alexithymia. There are also significant relationships between sensory processing sensitivity and alexithymia regardless of trauma. In a study examining the relationships between three factors of sensory processing sensitivity (easy arousal, low emotional threshold, aesthetic sensitivity) and alexithymia, autism symptoms, anxiety and depression [64], aesthetic sensitivity (SPS component) was found to be negatively correlated with externally oriented thinking, which is a symptom of alexithymia. Easy arousal (SPS) and low emotional threshold (SPS) were associated with recognizing and describing emotions among the symptoms of alexithymia. It has been observed that individuals with high aesthetic sensitivity have less outward-oriented thinking (the alexithymia component) [64]. The mediating role of sensory processing sensitivity for the relationship between childhood trauma and psychopathology was found to be significant. Findings demonstrate that the relationship between childhood trauma, sensory processing sensitivity and psychopathology is consistent with the relevant literature [50, 65]. Although there is no study investigating mediator role of sensory processing sensitivity in this relationship, Aron, Aron and Davies [66] pointed out that individuals with a high level of sensitivity who have a negative childhood experience have a higher tendency to be shy (social introversion/social withdrawal) and express negative affect (fearfulness, anxiety, depression). Also, Aron, Aron, and Jagiellowicz [67] stated that children with high levels of sensitivity in their childhood were highly reactive to positive and negative events. While a childhood environment, negative or traumatic experience may increase the individual’s sensitivity to sensory processing, individuals with a high level of processing sensitivity with childhood trauma may tend to experience psychological distress. Therefore, longitudinal studies are needed to explain the nature of this relationship.

Looking at the role of alexithymia, alexithymia mediates the relationship between childhood trauma and psychopathology. Studies have shown that alexithymia is associated with childhood trauma [54, 68, 69]. In a study comparing alexithymia and early life stresses in healthy samples with low and high alexithymia levels, a positive correlation was found between early emotional neglect and alexithymia [35]. In a study that examined the relationship between posttraumatic stress disorder (PTSD) and alexithymia, high levels of alexithymia were associated with childhood traumas (emotional and physical neglect) [70]. Studies have indicated that alexithymia is positively associated with psychopathology [68, 71]. For instance, studies examining the relationship between depression and anxiety and alexithymia reveal that high-level of depression and anxiety are associated with high-level of alexithymia [72, 73]. There are many studies that reveal or try to explain that alexithymia has a mediating role in the relationship between childhood trauma and psychopathology [68, 74]. Zou and his colleagues [71] emphasized that alexithymia mediates the relationship between certain types of childhood trauma and the severity of panic disorder. The presence of low expressions of the S or Lg allele of the 5-HTTLPR gene in individuals with childhood trauma was found to be associated with higher levels of alexithymia [75]. It is noteworthy that these genes [75] and alexithymia [69] are also associated with emotion regulation. From this point of view, it will be important to study emotion regulation difficulty in future studies. Considering that alexithymia is associated with both childhood trauma and psychopathology, it is thought that the level of alexithymia may be a risk factor or a predisposition factor.

In the mediation analysis, although the EYES test is not associated with childhood trauma, it is positively associated with psychopathology. However, although in the mediation analysis the EYES test is positively associated with psychopathology, the indirect effect of the EYES test on the relationship between childhood trauma and psychopathology is not significant. Accordingly, the EYES test results did not mediate the relationship between childhood trauma and psychopathology. Although rare, there are studies showing that childhood trauma and mentalization skills are not related [76]. Mostly, studies have demonstrated that childhood trauma is negatively related to mentalization skills and psychopathology [43, 77] In addition, research showed that mentalization skills mediate between childhood trauma and psychopathology [77, 78]. Many factors may have affected the results of the EYES test in this study. The mean of the participants’ age in this study is 30.65, 93% are university graduates, 78.4% are females. Most participants perceived their socio-economic levels are moderate (*N* = 198, 58.8%) and good (*N* = 111, 32.9%). Studies have indicated that females are better at recognizing emotions than male and young people compared to older individuals, and individuals have experienced a decrease in emotion recognition skills from the age of 30 [79, 80]. Predominantly young and female participants’ profile in our study may be a factor in explaining the weak relationship between the EYES test and psychopathology. It is considered that the participants with a high level of education can have better emotional and mental skills. In partial correlation (gender controlled) analysis, the EYES test was not associated with psychopathology. Clinical groups and clinically high-risk groups showed much lower emotion recognition performance compared to healthy control groups [81, 82]. In this study, the participants do not represent a clinical sample. Finally, it was thought that low reliability coefficient (α = .47) of the test may be a factor in the absence of expected relationships between reading mind in the EYES test, alexithymia and psychopathology. In this study, results of the EYES test were obtained from the online environment. Perception of the pictures in the computer environment may have created a problem. Also, the high number of female and higher education participants in our sample may have reduced the reliability of the scale. Since the reliability of the EYES test scale is also low, it should be taken into account that the findings obtained from the EYES test may not have been obtained from a reliable source.

The current limitations of the study were sampling characteristics and features of measurement tools. Although the sample size seems sufficient, the demographic characteristics of the participants are similar in terms of education and age. Most of the participants were composed of individuals who are young and higher education levels. The balance of men and women in the sample could not be achieved in this study. Additionally, the limited number of participants negatively affects the generalizability of the study results. The data in this study are based on self-reports of individuals. Although the confidentiality of the data is preserved, individuals may have difficulties in answering questions that may be difficult for them (e.g., childhood trauma scale). The high number of items in the scales we used in this study may have caused reluctance for participants to respond or may have caused random responses. Therefore, it seems more convenient that the scales that will be used in future studies are shorter and advantageous in time. In addition to self-report based measurement tools, objective measurement tools and experimental methods can be used. Also, people may misremember retrospective childhood trauma, and may be affected by their current emotional state in these recall situations. This situation may create limitations on the basis of retrospective data. The Cronbach Alpha value of the Brief Symptom Inventory used in the study was found to be 0.97. Cronbach alpha values of 0.58–0.97 are considered satisfactory. When there are reliability values close to 1, it requires careful use of the scale in the future [83]. This study is a cross-sectional study; therefore, it only provides information about the time the data was collected. Obtaining wide-ranging findings with longitudinal studies will be more effective in examining childhood trauma. Studies that follow-up from childhood or adolescence to adulthood may be important in order to better understand the effects of childhood trauma. Differences in psychopathology of people who were exposed to childhood trauma and those who were not can be tested in subsequent studies. Since the study is also a correlational study, it is not possible to establish a cause-effect relationship between concepts. Another important limitation of this study was that the scales were always presented in the same order. Results may have been affected due to the order in which the scales were presented. It is recommended to pay attention to this in future studies. The Childhood Trauma Scale was the last scale presented. Participants may feel negative emotions after completing this scale. To prevent this, researchers could use a scale with a more positive ending. As another option, participants could be asked to contact researchers when they are uncomfortable. There should have been more ethical precautions for this study.

As a result, although this study has certain limitations, many scales have been studied with a good number of samples. It suggests that we contribute to limited literature by working on sensory processing sensitivity and reading mind in the EYES test. The study model we have established and results showed that sensory processing sensitivity and alexithymia played a mediator role in the relationship between childhood traumas and psychopathology. Our study may contribute to the understanding of what may lead to a person’s vulnerability to experiencing psychopathology after childhood trauma. This study is an important step that identifying these connections is imperative in digging a deeper understanding for factors from trauma that mediate or contribute to psychopathology. It is suggested that future studies find ways to take a deeper dive into interoception to start getting at the neurobiological underpinnings of regulation challenges for individuals with trauma. Since the model we have established is a mediation model, it cannot give us information about the nature of this relationship. As a clinical perspective, it is recommended to conduct prospective longitudinal follow-up studies in order to test the model we have established in adolescents and young adults with trauma. Comparisons based on measurement of HPA activity in adults exposed to childhood trauma (neuroendocrine variables, cortisol level, etc.) and associations with psychopathology may contribute to the literature. When childhood trauma is evaluated in future studies, it is recommended to consider the neuroendocrine variables of the participants in cooperation with different disciplines. Genetically, if the HPA axis has a less responsive structure, this situation is protective in the effect of early negative life events on emotion recognition [84]. Also, studies on the relationship of early childhood parental care with the HPA axis indicate that sensitive and responsive care and secure attachment are buffers for the HPA axis [85]. In future studies, the examination of genetic factors and different protective factors may contribute to the clarity of the study.

## Conclusion

The extent to which childhood trauma affects psychological health in adulthood is frequently examined today, and the importance of intervening in childhood trauma is emphasized. For this reason, it is crucial to examine in detail the strong relationship between childhood trauma and psychopathology and to understand in which areas and how to intervene. Sensory processing sensitivity and alexithymic characteristics of individuals can be examined in the treatment of psychological problems of individuals who have experienced childhood trauma. Investigation of these characteristics and development of intervention programs for individuals with childhood trauma enable more comprehensive prevention and intervention programs in terms of psychopathology that may arise in the future.

## Data Availability

The datasets generated and/or analyzed during the current study are not publicly available due to confidentiality, but are available on reasonable request from the corresponding author. The set of questionnaires used in this study only included published questionnaires (BSI, CTQ, EYES, TAS-26 see methods section for details).

## Abbreviations

BSI: Brief Symptom Inventory
CTQ: Childhood Trauma Questionnaire
EYES: Reading mind in the eyes test
HPA: Hypothalamic-pituitary-adrenal axis
PTSD: Post-traumatic stress disorder
SPS: Sensory Processing Sensitivity Scale
TAS-26: Toronto Alexithymia Scale
